# Ruptured Cesarean Scar Pregnancy: A Case Report

**DOI:** 10.31729/jnma.4465

**Published:** 2019-06-30

**Authors:** Prishita Shah, Rosina Manandhar, Meena Thapa, Rachana Saha

**Affiliations:** 1 Departmentof Obstetrics and Gynecology, Kathmandu Medical Collegeand Teaching Hospital, Sinamangal, Kathmandu, Nepal

**Keywords:** *cesarean*, *hysterectomy*, *maternal mortality*, *pregnancy*, *scar*, *uterine rupture*

## Abstract

Cesarean scar pregnancy is a rare variant of ectopic pregnancy where the fertilized ovum gets implanted in the myometrium of the previous cesarean scar. The incidence of Caesarean Scar Pregnancy among ectopic pregnancies is 6.1% and it is seen in approximately 1 in 2000 normal pregnancies. As trophoblastic invasion of the myometrium can result in uterine rupture and catastrophic hemorrhage termination of pregnancy is the treatment of choice if diagnosed in the first trimester. Expectant treatment has a poor prognosis and may lead to uterine rupture which may require hysterectomy and subsequent loss of fertility. We present a case report of a 24-year-old female G_2_P_1_L_1_with ruptured cesarean scar pregnancy who underwent emergency laparotomy and subsequently hysterectomy. In this case report, we aim to discuss ruptured cesarean scar pregnancy as obstetric emergency and methods by which we can make an early diagnosis that it can be managed appropriately so as to prevent maternal morbidity and mortality.

## INTRODUCTION

Caesarean scar pregnancy (CSP) is a rare variant of ectopic pregnancy where the fertilized ovum gets implanted in the myometrium of the previous cesarean scar. Incidence of CSP among ectopic pregnancies is 6.1% and seen in approximately 1 in 2000 normal pregnancies.^1^ Recent rise in incidence is believed to be due to increased rate of cesarean delivery.^2^ It is associated with maternal morbidity, secondary infertility, and mortality. Diagnosis can be achieved through transabdominal and TVS, color Doppler and MRI.^3^ In this case report, we aim to discuss one such case of a ruptured CSP which presented as an obstetric emergency, how it can be prevented in the future with appropriate and timely diagnosis.

## CASE REPORT

A 24-year-old female G_2_P_1_L_1_ at 13 weeks of gestation with one previous LSCS was brought in semi-conscious state in our hospital. She was hyportensive and tachycardic. According to her husband she was pregnant and has taken abortificant drug after confirming pregnancy over a counter 1 month back. Since then she had PV bleeding which was minimal, soaking 1-2 pad per day and has resolved spontaneously few days ago. On examination she was in state of shock, BP was unrecordable, peripheral pulses were non-palpable. Carotid pulse was feeble and rapid with the rate of 165bpm. Per abdomen examination revealed distention of whole abdomen with tenderness and rigidity. On per speculum examination, bleeding was seen. Uterus size could not be assessed due to tenderness but both fornices were found to be full and tender per vaginally. Resuscitation was started immediately. All investigations were normal except Hb of 6.8 gm%. Bedside USG showed intrauterine pregnancy with massive free fluid in peritoneal cavity with fetus in lower pole of uterus. With the view of massive hemoperitonium emergency laparotomy was performed.

**Figure 1.**
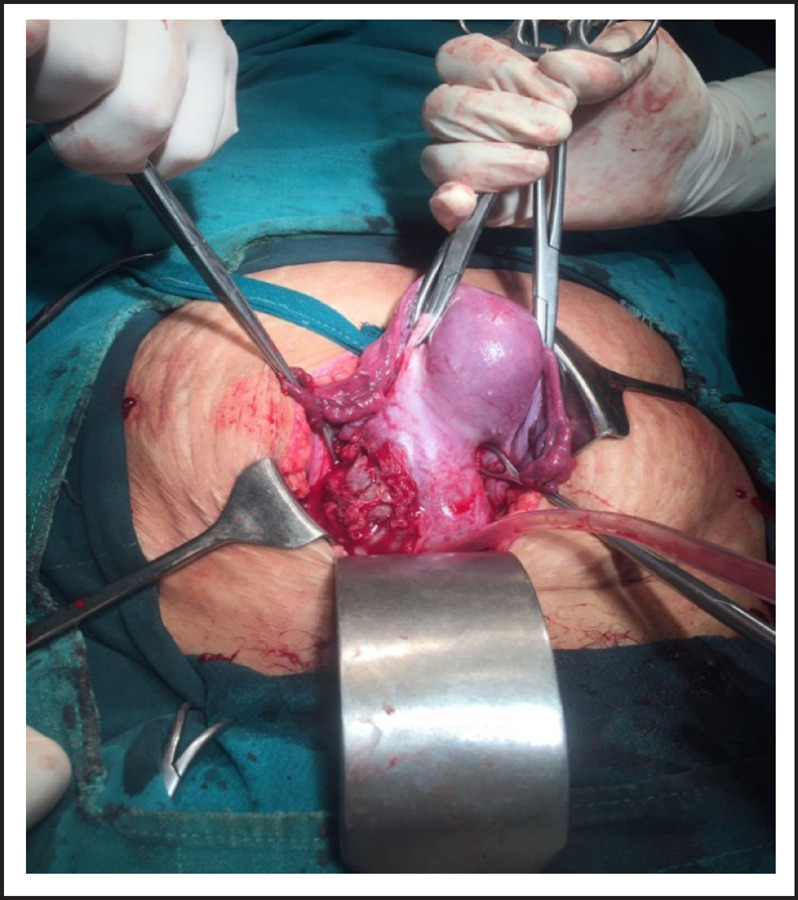
Rent of 2x3cm on right edge of of previous scar.

**Figure 2.**
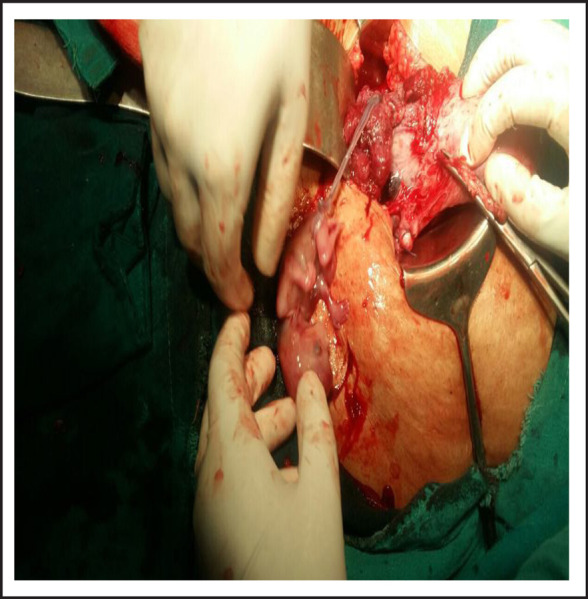
Fetus of around 12 weeks removed from rupture site.

**Figure 3.**
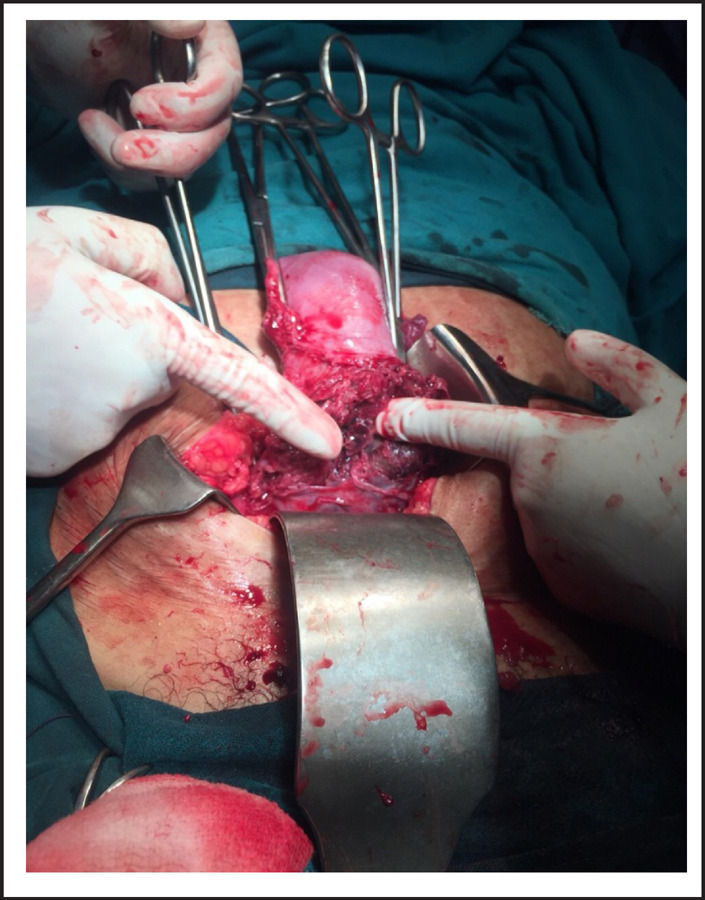
Placental tissue embedded on myometrial scar.

**Figure 4.**
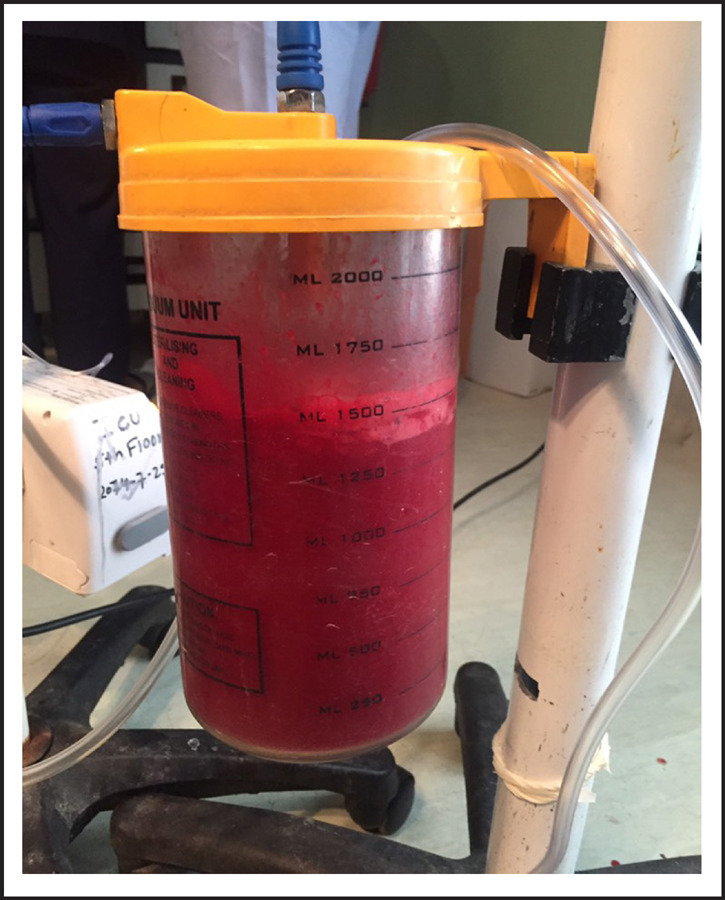
Blood loss.

On laparotomy, there was massive hemoperitoneum of around three liters. Both tubes and ovaries were found normal. Uterus was 12 weeks size with distension at lower segment. There were big clots at uterovesicle pouch and on removing it, there was rent of 3x2cm at right edge of previous scar with active bleeding from that site through which gestational sac with fetus was visualized. Whole of myometrium around the rupture site was embedded by placental tissue. The sac along with fetus was removed. Uterine artery was ligated and repair of rent was tried but bleeding could not be controlled so subtotal hysterectomy was performed. Blood transfusion was started during surgery. She was transferred to ICU intubated state. Total four units of whole blood and two pint fresh frozen plasma, two pint PRP transfused. She was extubated next day and stayed there for two more days. She recovered and was discharged on her 5^th^ post-operative day.

## DISCUSSION

CSP was first reported in 1978 by Laren and Solomon.^4^ Since then more than 751 cases have been reported^5^ and incidence is rising due to the ever increasing cesaerean rates. Maymon et al. reported an interesting association between cesarean deliveries for breech and subsequent scar pregnancies which was a case specific history in our case.^6^ Almost all CSPs are terminated till the second trimester, very few of these pregnancies reported in literature have progressed beyond first trimester. It is likely that if a developing pregnancy in a caesarean section scar were to continue to the second or third trimesters, there would be a substantial risk of uterine rupture with catastrophic haemorrhage with a high risk of hysterectomy causing serious maternal morbidity and loss of future fertility which was what happened in our case. Besides this, there is also a danger of invasion of the bladder by the growing placenta and any pregnancy that protrudes through the scar, if viable, can implant on other abdominal organs and continue to grow as a secondary abdominal pregnancy^7^which in turn has its own set of complications. Little information is available on the natural history of this condition. About 13% of the CSP cases are either missed or misdiagnosed and these are the cases which present late and with significant morbidity. So, focus should be channeled towards timely diagnosis. TVS on its own has a diagnostic sensitivity of 86.4%.The recommended approach for reliable diagnosis of CSP includes: use of transvaginal USG probe at 5-12 MHz, visualization of an empty uterine cavity as well as an empty endocervical canal, detection of the placenta and/or gestational sac embedded in the hysterectomy scar, in early gestations (less than eight weeks) a triangular gestational sac that fills the niche of the scar - in later gestations (after eight weeks) this shape may become round or oval, a thin (1-3mm) or absent myometrial layer between the gestational sac and the bladder and the presence of a prominent vascular pattern at or in the area of a hysterectomy scar in the presence of a positive pregnancy test.^8^

Magnetic resonance imaging (MRI) can be used as an adjunct to the ultrasound scan if TVS is inconclusive. Laparoscopy or laparotomy may be performed if TVS or MRI fails to identify CSP.^9^ No specific guidelines available for the management of CSP, diDerent treatment methods of CSP ranging from medical to surgical or sometimes, a combination of these has been proposed by different author. The treatment method is selected on the basis of clinical presentation and treating clinician's skill and experience. The treatment method is selected on the basis of clinical presentation, clinician's skill and experience. Wang et al. treated 71 patients with methotrexate therapy (local or IM) with or without suction curettage and concluded that both could treat majority of CSP successfully, but the combination has better outcome.^10^ Salomon et al. reported the first case of heterotopic pregnancy which was first successfully treated with USG guided KCL injection.^11^ Li et al. evaluated 124 CSP and treated with three different modalities and concluded that UAE with hysteroscopy to be most efficient but in all these cases patients presented at early stage.^12^ In our case patient presented with hypovolemic shock with rupture ectopic at scar site. We did laparotomy with evacuation of products and repair of uterus was not possible so end up with hysterectomy. Similar case was reported by Nankali et al. and Singh et al. and the same treatment approach was adopted but could able to save uterus.^2,13^ In spite of multiple challenges during diagnosis and treatment, recovery was made possible. Even though this pregnancy was unwanted for her however regret is definitely there for not been able to save her uterus. The lesson to be learned from this case is the golden role of: clinical judgment, high index of suspicion, multidisciplinary approach, for the treatment. In background of massive hemoperitoneum with positive UPT we must always think about of CSP, though incidence is rare, as possible differential diagnosis.

## Consent

**JNMA Case Report Consent Form** was signed by the patient and the original is attached with the patient's chart.

## Conflict of Interest

**None.**
